# The effectiveness of traditional vs. velocity-based strength training on explosive and maximal strength performance: A network meta-analysis

**DOI:** 10.3389/fphys.2022.926972

**Published:** 2022-08-10

**Authors:** Steffen Held, Kevin Speer, Ludwig Rappelt, Pamela Wicker, Lars Donath

**Affiliations:** ^1^ Department of Intervention Research in Exercise Training, German Sport University Cologne, Cologne, Germany; ^2^ Department of Sports Science, Bielefeld University, Bielefeld, Germany

**Keywords:** power-based training, maximum strength, counter movement jump, squat jump, mean concentric velocity

## Abstract

This network meta-analysis aimed at evaluating the effectiveness of different velocity-based (VBT) and traditional 1RM-based resistance training (TRT) interventions on strength and power indices in healthy participants. The research was conducted until December 2021 using the online electronic databases PubMed, Web of Science, PsycNet, and SPORTDiscus for studies with the following inclusion criteria: 1) controlled VBT trials, 2) strength and/or jump and/or sprint parameters as outcomes (c), participants aged between 18 and 40 years, and 4) peer-reviewed and published in English. Standardized mean differences (SMD) using a random effects models were calculated. Fourteen studies with 311 healthy participants were selected and 3 networks (strength, jump, and sprint) were achieved. VBT, TRT, repetitions in reserve (RIR), low velocity loss (lowVL), and high velocity loss (highVL) were ranked for each network. Based on P-score rankings, lowVL (P-score ≥ 0.59; SMD ≥ 0.33) and highVL (P-score ≥ 0.50; SMD ≥ 0.12) revealed favorable effects on strength, jump, and sprint performance compared to VBT (P-score ≤ 0.47; SMD ≤0.01), TRT (P-score ≤0.46; SMD ≤ 0.00), and RIR (P-score ≤ 0.46; SMD ≤ 0.12). In conclusion, lowVL and highVL showed notable effects on strength, jump, and sprint performance. In particular for jump performance, lowVL induced favorable improvements compared to all other resistance training approaches.

## Introduction

Resistance training is considered an important part of an athlete’s weekly training schedule in both individual and team sports (Healy et al., 2019; Drury et al., 2021). Besides morphological and functional adaptations at the muscular level (Grgic et al., 2021), relevant performance abilities such as strength, jumping, and sprinting ability relevantly benefit from adequate resistance training (Suchomel et al., 2016). Traditional resistance training mainly relies on training approaches based on a certain percentage of the one repetition maximum (1RM) or autoregulative (perceived exertion or reps in reserve) (Zourdos et al., 2016).

Within the last decades, velocity-based resistance training (VBT) as an alternative strength training approach has gained increasing attention (González-Badillo and Sánchez-Medina, 2010). During VBT the training intensity is controlled by monitoring the mean concentric velocity (MCV) of each repetition (González-Badillo and Sánchez-Medina, 2010) using inertial sensors (Held et al., 2021b) or linear position transducers (Held et al., 2021a). In general, the level of fatigue increases gradually as a function of increasing effort during a training set (Sánchez-Medina and González-Badillo, 2011). Therefore, a decreasing MCV across repetitions within a set provides a feasible, simple, and promising tool to clearly objectify levels of fatigue (Sánchez-Medina and González-Badillo, 2011). Hence, VBT allows the application of a homogeneous stimulus across individuals (Pareja-Blanco et al., 2020) and resistance training control without excessive exhaustion (Padulo et al., 2012). Further, employing the “two-point method” (García-Ramos et al., 2018), the load-velocity relationship enables an athlete’s 1RM prediction without applying maximum loads. Compared to traditional 1RM-based strength training with large within subject day-to-day variability (Kiely, 2012; Jovanović and Flanagan, 2014), this relationship has been reported to be load- and exercise-specific (Beck et al., 2020), but robust over long-term training progress (González-Badillo and Sánchez-Medina, 2010).

The term VBT covers a variety of approaches (Weakley et al., 2021), with the two main approaches employing velocity zones (i.e., completing a set of repetitions within a pre-defined individual or generic velocity range) or velocity loss thresholds (i.e., performing repetitions within a set until the velocity drops below a predefined threshold) (Orange et al., 2022). In this context, qualitative assessment of the literature in two recently published systematic reviews (Włodarczyk et al., 2021; Baena-Marín et al., 2022) indicates superior adaptational potential in terms of 1RM, sprinting, and jumping performance for VBT approaches employing a velocity loss threshold of 10–20%. Therefore, combining different VBT approaches (e.g., employing velocity zones and velocity loss thresholds) to allow comparison with traditional strength training methods in the context of a meta-analysis (Liao et al., 2021; Orange et al., 2022) may impede well-informed decision making for trainers, athletes, and practitioners in the field of velocity-based strength training due to the partial heterogeneity of the studies (Liao et al., 2021).

Hence, a network meta-analysis (NMA) that enables effect size estimation based on both direct and indirect study comparison can serve as a proper alternative to synthesize available evidence in the field of velocity-based strength training. Specifically, this method allows estimating comparative effects of treatment arms that have not been directly compared in randomized trials (Caldwell et al., 2005). Thus, employing a NMA allows the comparison of VBT approaches with different velocity loss thresholds or velocity zones against traditional strength training methods. Therefore, the present network meta-analysis aimed at examining and comparing the effects of different strength training interventions by distinguishing between traditional and velocity-based training approaches based on maximal and explosive performance indices. We hypothesized that the VBT approaches in general might lead to superior effects on relevant performance surrogates compared to traditional 1RM-based training. We furthermore assume that limiting the loss of MCV to a lower limit will improve speed strength performance adaptations. The overall results of this NMA might help coaches and researchers to better select their training regime based on the intended training adaptations.

## Materials and methods

### Search and screening procedures

This review was conducted in accordance with Preferred Reporting Items for Systematic Reviews and Meta-Analyses for Network Meta-Analyses (PRISMA-NMA) (Hutton et al., 2015). The literature search and screening were independently conducted by two researchers (KS and HB). Four health-related, biomedical, and psychological databases (PubMed, Web of Science, PsycNet and SPORTDiscus) were screened from inception of the respective journal until December 6, 2021. Relevant search terms (operators) were combined with Boolean conjunctions (OR/AND) and applied on two search levels (Table1).

**TABLE 1:** Search strategy.

Search level	Search terms with Boolean operators
Search #1	“velocity based training” OR “velocity based” OR “vbt” OR “concentric velocity” OR “mean concentric velocity” OR “movement velocity” OR “barbell velocity” OR “velocity loss” OR “power based training”
Search #2	#2 AND (”1 repetition maximum” OR “1RM” OR “one repetition maximum” OR “MVC” OR “muscle strength” OR “muscular strength” OR “hypertrophy” OR “muscle hypertrophy” OR “muscular hypertrophy” OR “muscle fibre” OR “muscle fiber” OR “muscle thickness” OR “CSA” OR “cross-sectional area” OR “muscle size” OR “girth” OR “torque” OR “rate of torque development” OR “RTD” OR “rate of force development” OR “RFD” OR “strength development rate” OR “SDR” OR “jump” OR “drop jump” OR “depth jump” OR “DJ” OR “counter movement jump” OR “CMJ” OR “vertical jump”)

The researchers also tracked cited articles and hand searched relevant primary articles and reviews. Duplicates were removed and the remaining studies underwent a manual screening. They were gradually screened using 1) the titles, 2) abstracts, and 3) full-texts of the potentially eligible articles. The final decision for inclusion or exclusion was made by two independent researchers (KS and SH). The following inclusion criteria based on the PICOS approach [population (P), intervention (I), comparators (C), main outcome (O), and study design (S)] were used: Full-text article published in English in a peer-reviewed journal; participants were healthy adults between 18 and 40 years (P), without any cognitive, neurological, orthopedic, and/or cardiac conditions that could affect physical testing and training; velocity-based training served as the interventional strategy of interest (I); active control group(s) that performed non velocity or velocity loss based resistance training and/or inactive control group(s) that did not receive any intervention served as a comparator (C); at least one outcome of strength (1RM), jump (CMJ or SJ), and sprint (10–30 m) performance had to be evaluated in the study (O); and prospective two- or multi-armed controlled intervention study with pre- and post-testing and more than 7 days duration (S). The exclusion criteria were: No adequate control conditions, which made integration into the network impossible.

### Assessment of the methodological quality of included studies

The methodological quality (including risk of bias) of the included studies was independently rated using the PEDro (Physiotherapy Evidence Database) scale (Maher et al., 2003). The PEDro scale contains 11 dichotomous (yes or no) items, in which the criteria 2–9 rate randomization and internal validity and the criteria 10–11 rate the presence of statistically replicable results. Criterion 1 relates to external validity, but will not be used to calculate the PEDro score. Studies were rated independently by two non-blinded reviewers (KS and HB) who needed to obtain consensus on every item. Discordant study ratings were discussed point to point by two reviewers (KS and SH) who then came to a decision. To represent a high-quality study, the PEDro score had to be ≥6 on a scale from 0 to 10 (Maher et al., 2003).

### Data extraction

Relevant data (required for calculating effect sizes) were extracted independently by two researchers (KS and SH) using a standardized extraction Excel spreadsheet adapted from the Cochrane Collaboration (Higgins et al., 2022). Means and standard deviations of pre-test and post-test scores on all strength, jump, and sprint related tests were extracted, along with the number of participants assessed in each group. If these point and variability measures were not reported in the full-text article, either the means and pooled within-group standard deviations of change scores were entered in an electronic spreadsheet or the authors of the article were contacted, and missing values were requested three times over the course of 2 months. If studies only presented means and standard deviations in figures, the WebPlotDigitizer Version 4 (Free Software Foundation, Boston, MA, United States) was used to extract means with standard deviations. For studies with multiple outcomes for a single neuromuscular test, the condition with the highest demand (as determined by two researchers) on the respective domain was extracted. In the case of multiple relevant neuromuscular tests, effect sizes and standard errors were pooled. Subsequently, all neuromuscular tests and outcomes were categorized into strength, jump, and sprint. In addition to these outcomes, relevant study information regarding author, year, number of participants, interventional data (weeks, frequency, duration per session, type of intervention), control condition, and PEDro scale scores were also recorded. For the simplification of the networks, similar treatments have been summarized into low velocity loss (LowVL), high velocity loss (HighVL), repetitions in reserve-based training (RIR), velocity-based resistance training (VBT), and traditional 1RM based resistance training (TRT). Thereby, LowVL and HighVL were defined as velocity losses of ≤20% and >20%, respectively.

### Statistics

The standardized mean difference (SMD) and its 95% confidence intervals (95CI) were calculated for all the interventional treatments as a measure of treatment effectiveness. Thereby, SMDs were calculated as differences between means divided by the pooled standard deviations (trivial: SMD <0.2, small: 0.2 ≤ SMD <0.5, moderate: 0.5 ≤ SMD <0.8, large SMD ≥0.8) (Cohen, 1988). Subsequently, three separate network models for strength, sprint, and jump were computed. In order to visualize the networks, a network graph was created for each of the three networks. The estimations of treatment effects were calculated based on a random effects model (Senn, 2007). TRT was defined as reference treatment for the following network meta-analytical procedure. A ranking was created based on the P-score of the individual treatments. The P-score calculation following the surface under cumulative ranking (SUCRA) protocol of a treatment is obtained from estimating the effect sizes of pairwise treatment comparisons and presuming their point estimates are normally distributed. P-scores range from 0 to 100%, with 0 or 1 being the theoretically worst and best treatment, respectively, (Rücker and Schwarzer, 2015). P-scores in a frequentist NMA are analogous to the SUCRA (Mbuagbaw et al., 2017) values found in Bayesian NMA (Rücker and Schwarzer, 2015). Additionally, a forest plot was created to further visualize the ranking and effects of the treatments. The decomposed Q-statistics (within and between designs) were used to assess potential heterogeneity and inconsistency. Heterogeneity and inconsistency were further quantified by I^2^ (Higgins and Thompson, 2002). Funnel plots were created to check potential publication bias, whereby Egger’s test for asymmetry of the funnel plot (Egger et al., 1997) was used. R software (version 4.1.1; The R Foundation for Statistical Computing) and the package “netmeta” (Rücker et al., 2021) was used for all calculations and figures.

## Results

### Trial flow

**FIGURE 1:**
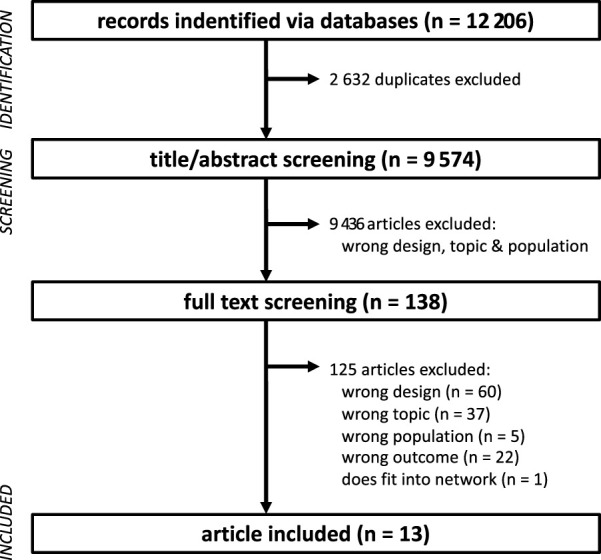
Flow chart of the different phases of study screening and selection.

In total 12,206 potentially relevant articles were initially found (Figure 1). After removing duplicates, 9,574 article titles and abstracts were carefully screened for relevance. The full-texts of the remaining 138 potentially relevant articles were thoroughly studied. Altogether, 125 papers were excluded as they did not meet the inclusion criteria or fulfilled the exclusion criteria. Consequently, quantitative and qualitative data were extracted from a final set of 13 articles (Table 2).

**TABLE 2:** Overview of the included studies.

Study	Population	Sample [n]	Age [yrs]	Training age [y]	Duration [w]	Sessions [n/wk]	Outcome	Interventions
Andersen et al. (2021)	resistance trained males	10	23.0 ± 4.3	4.5	9	2	1RM leg press	**LowVL**: Velocity Based Resistance Training with 15% Velocity Loss, volume matched, intravidual leg comparison
								**HighVL**: Velocity Based Resistance Training with 30% Velocity Loss, volume matched, intravidual leg comparison
Banyard et al. (2021)	resistance trained males	24	25.5 ± 5.0	>2	6	3	1RM squat, CMJ, sprint (10m, 20 m)	**VBT:** Velocity Based Resistance Training; with progression; 25 reps per session
								**TRT:** Traditional 1RM Based Resistance Training, progression from 59 to 85% 1RM; 25 reps per session
Dorrell et al. (2020)	resistance trained males	16	22.8 ± 4.5	>2	6	2	1RM squat, CMJ	**VBT:** Velocity Based Resistance Training via velocity zones; 12 to 32 reps per exercise and session; 70–90% 1RM squat, benchpress, overhead press, deadlift
								**TRT:** Traditional 1RM Based Resistance Training, load matched to VBT; 12 to 32 reps per exercise and session; 70–90% 1RM squat, bench press, overhead press, deadlift
Galiano et al. (2020)	physically active male	28	22.1 ± 2.9	>1.5	7	2	1RM squat, CMJ, sprint (20 m)	**LowVL:** Velocity Based Resistance Training with 5% Velocity Loss, at fixed 1.14 m/s; total reps 156.9 ± 25.0; 50% 1RM squat
								**HighVL:** Velocity Based Resistance Training with 20% Velocity Loss, at fixed 1.14 m/s; total reps 480.5 ± 162.0; 50% 1RM squat
Held et al. (2021a)	rowers	21 (4 females)	19.6 ± 2.0	>2	8	2	1RM Squat	**LowVL:** Velocity Based Resistance Training with 10% Velocity Loss; total reps 2,145 ± 285; 80% 1RM squat, deadlift, bench row and bench press
								**TRT:** Traditional 1RM based training to repetition failure; total reps 2,825 ± 100; 80% 1RM squat, deadlift, benchrow and bench press
Jiménez-Reyes et al. (2021)	physically active males	24	23.1 ± 4.2	>2	8	2	1RM squat, CMJ, sprint (10m, 20 m)	**VBT:** Velocity based Training, load matched; 6 to 32 rpes per exercise and session; 50–80% 1RM squat
								**TRT:** Traditional 1RM based Resistance Training; 6 to 32 rpes per exercise and session; 50–80% 1RM squat
Montalvo-Perez et al. (2021)	Female cyclists	17 (17 females)	26.0 ± 7.0	>2	6	2	1RM squat	**VBT:** Velocity Based Resistance Training, at about 65% 1RM; 3 sets per exercise, squat, hip thrust and lunges
								**TRT:** Traditional 1RM based Resistance Training, with progression from 80–90% 1RM; 12 to 32 reps per exercise and session, squat, hip thrust and lunges
Pareja-Blanco et al. (2017b)	male soccer players	16	23.8 ± 3.5		6	3	1RM squat, CMJ, sprint (30 m)	**LowVL:** Velocity Based Resistance Training with 15% Velocity Loss; total reps 251.2 ± 55.4; 50–70% 1RM squat
								**HighVL:** Velocity Based Resistance Training with 30% Velocity Loss; total reps 414.6 ± 124.9; 50–70% 1RM squat
Pareja-Blanco et al. (2020)	resistance trained male	55	24.1 ± 4.3	1.5-4	8	2	1RM Squat	**LowVL:** Velocity Based Resistance Training with 0/10% Velocity Loss; total reps 143.6 ± 40.2; 70–85% 1RM
								**HighVL:** Velocity Based Resistance Training with 20/40% Velocity Loss; total reps 237.1 ± 64.6; 70–85% 1RM
Perez-Castilla et al. (2018)	physically active males	20	22.1 ± 2.1	>2	4	2	1RM loaded jump, CMJ, sprint (15 m)	**LowVL:** Velocity Based Resistance Training with 10% Velocity Loss, volume matched, drop jump, counter movement jump and deadlift up to 50% body mass
								**HighVL:** Velocity Based Resistance Training with 20% Velocity Loss, volume matched, drop jump, counter movement jump and deadlift up to 50% body mass
Rodriguez-Rosell et al. (2020)	physically active males	25	22.8 ± 3.1	1-3	8	2	1RM squat, CMJ, sprint (10m, 20 m)	**LowVL:** Velocity Based Resistance Training with 10% Velocity Loss; total reps 109,6 ± 12.0; 70–85% 1RM squat
								**HighVL:** Velocity Based Resistance Training with 30% Velocity Loss; total reps 228.0 ± 76.6; 70–85% 1RM squat
Rodriguez-Rosell et al. (2021)	physically active males	35	21.6 ±2.8	1-3	8	2	1RM squat, CMJ, sprint (10m, 20 m)	**LowVL_** Velocity Based Resistance Training with 10% Velocity Loss; total reps 180.8 ± 29.0; 55–70% 1RM squat
								**HighVL:** Velocity Based Resistance Training with 30/45% Velocity Loss; total reps 429.5 ± 84.6; 55–70% 1RM squat
								**TRT:** 1RM based resistance training with progression from 50–70% 1RM; 55–70% 1RM squat
Shattock and Tee, (2020)	male rugby players	20	22.0 ± 3.0	>2	6	3	1RM squat, CMJ, sprint (10m, 20 m)	**VBT:** Velocity Based Resistance Training, load matched; total volume 149 270 ± 17 413 kg; 70–90% 1RM squat, bent. Over row, shoulder press, pull ups, benchpress, deadlift
								**RIR:** Reps in reserve (RIR) based resistance training, load matched; total volume 153 395 ± 13 574 kg; 70–90% 1RM squat, bent. Over row, shoulder press, pull ups, benchpress, deadlift

LowVL: low velocity loss (≤20%); HighVL: high velocity loss (>20%); RIR: repetitions in reserve based training; VBT: velocity-based resistance training; TRT: traditional 1RM based resistance training (TRT).

### Study characteristics and quality

**FIGURE 2:**
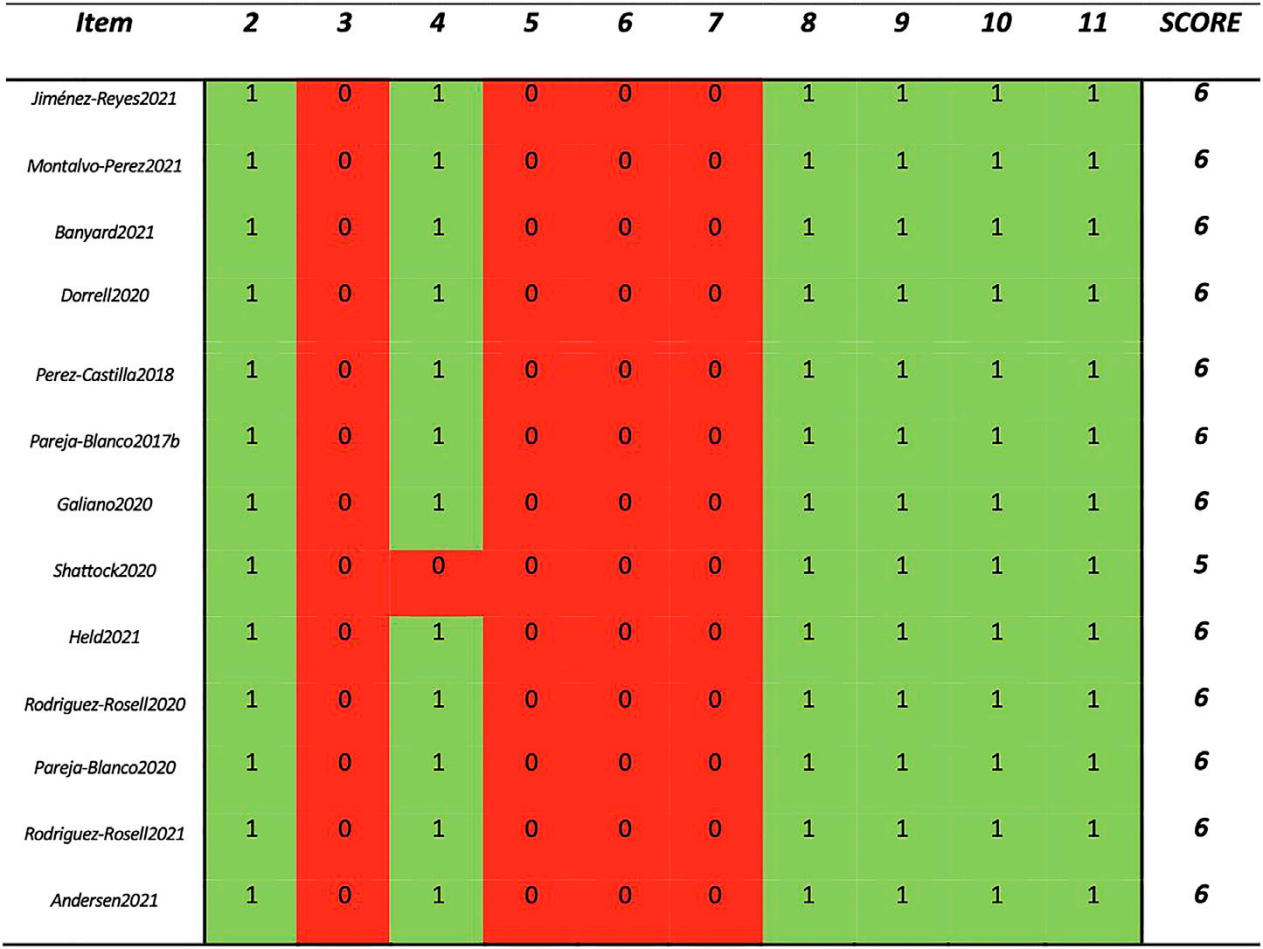
PEDro Score of each included study.

Included trials (311 healthy adults) consisted of 24 ± 11 participants per study (range 10 to 55) with an average age of 23.0 ± 1.7 years (range 19.6 to 26.0 years). The average study quality was high as indicated by a PEDro score of 5.9 ± 0.3 (range 5 to 6; Figure 2). Apart from one three armed design (Rodríguez-Rosell et al., 2021), most studies employed a two-armed design (Pareja-Blanco et al., 2017b; Pérez-Castilla et al., 2018; Dorrell et al., 2020; Galiano et al., 2020; Pareja-Blanco et al., 2020; Rodríguez-Rosell et al., 2020; Shattock and Tee, 2020; Held et al., 2021a; Andersen et al., 2021; Banyard et al., 2021; Jiménez-Reyes et al., 2021; Montalvo-Pérez et al., 2021).

### Strength, jump, and sprint network

**FIGURE 3:**
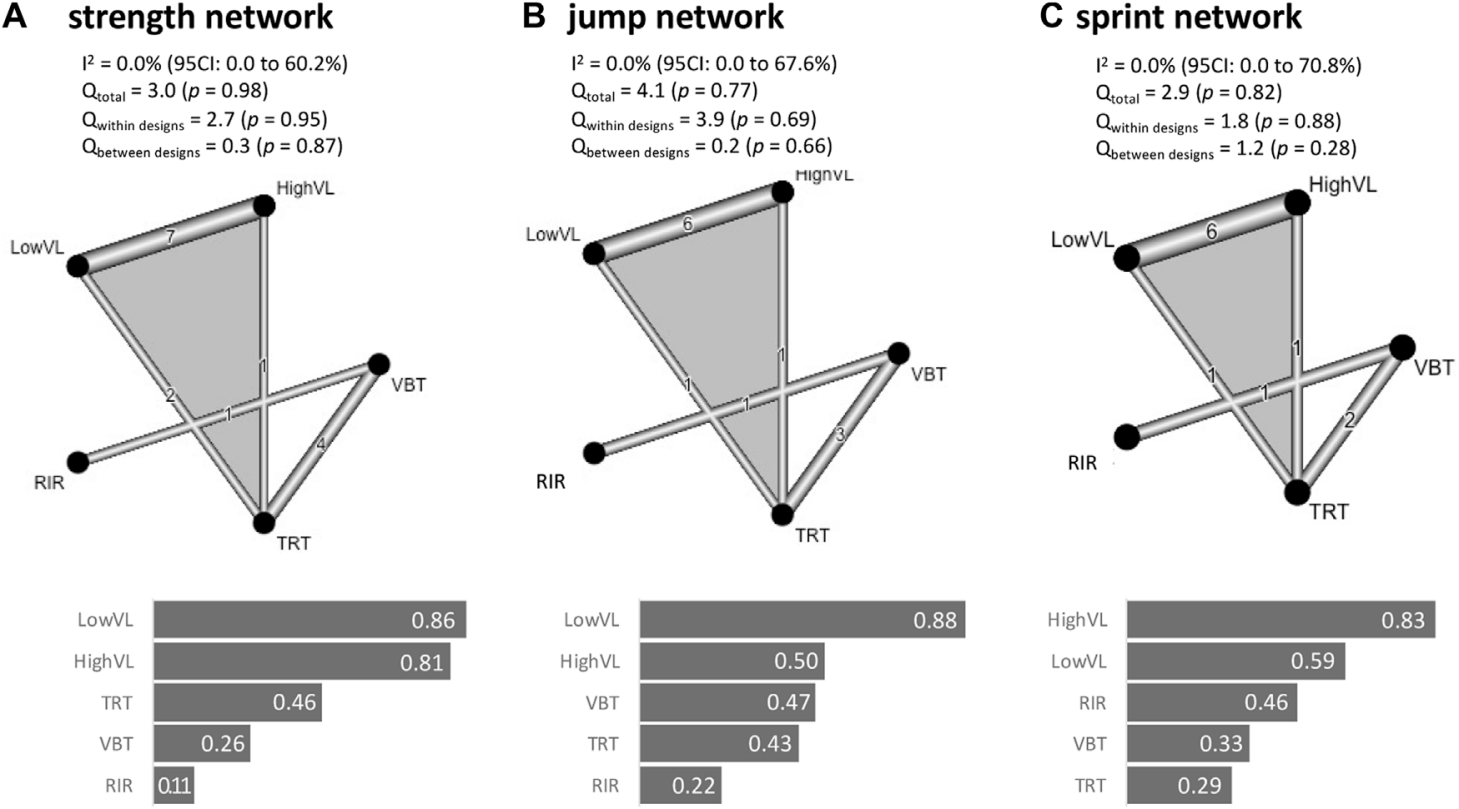
Network plots of the strength **(A)**, jump **(B)**, and sprint **(C)** network. In addition, I^2^, Q statistic, and P-score rankings are given. LowVL: low velocity loss (≤20%); HighVL: high velocity loss (>20%); RIR: repetitions in reserve-based training; VBT: velocity-based resistance training; TRT: traditional 1RM based resistance training (TRT).

**FIGURE 4:**
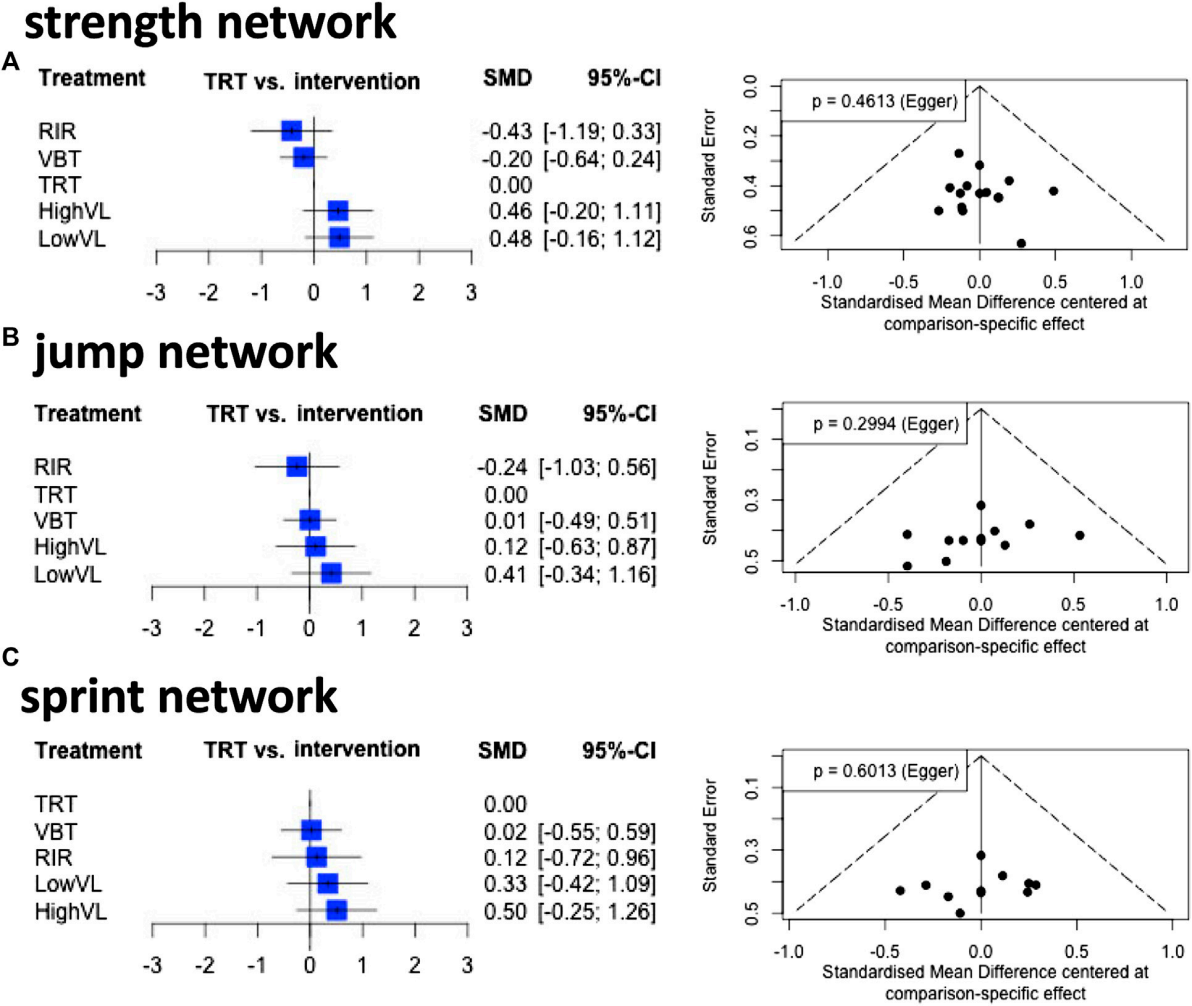
Forest and funnel plots for the strength **(A)**, jump **(B)**, and sprint **(C)** network. In addition, Egger´s p scores are given. LowVL: low velocity loss (≤20%); HighVL: high velocity loss (>20%); RIR: repetitions in reserve-based training; VBT: velocity-based resistance training; TRT: traditional 1RM based resistance training (TRT).

The strength, jump, and sprint networks revealed low heterogeneity and non-significant heterogeneity inconsistency (I^2^ and Q statistics; Figure 3). The funnel plot evaluations and non-significant Egger’s tests revealed no risk of bias for the strength, jump, and sprint network (Figure 4). Figures 2, 3 visualize the ranking of treatments and the pairwise comparison, respectively. In the strength network (Figure 3A), data from 13 studies (276 participants) representing 15 (pairwise comparison) effect sizes were included. The most common comparison was LowVL vs. HighVL (*n* = 7), followed by VBT vs. TRT (*n* = 4), and LowVL vs. TRT (*n* = 2). The jump network (Figure 3B) is based on 10 studies (220 participants) representing 10 (pairwise comparison) effect sizes. The most common comparison was LowVL vs. HighVL (*n* = 6), followed by VBT vs. TRT (*n* = 3). The sprint network (Figure 3C) contained data from 9 studies (204 participants), representing 9 (pairwise comparison) effect sizes. The most common comparison was LowVL vs. HighVL (*n* = 6), followed by VBT vs. TRT (*n* = 2).

## Discussion

This is the first network meta-analytical review that investigated and compared the effects of different velocity-based resistance training approaches with traditional 1RM-based resistance training settings. Interestingly, (high and low) velocity loss based resistance training approaches ranked (based on P-scores) consistently at the top two for the strength, jump, and sprint network, respectively. All three networks revealed small to moderate positive effects for both high and low velocity-loss-based resistance training approaches compared to a traditional resistance training approach. Other velocity based resistance training approaches ranked consistently between third and fourth place according to the P-score ranking of the strength, jump, and sprint network. Thereby, these other velocity based resistance training approaches revealed small negative to trivial positive effects on strength, jump, and sprint performance. Traditional 1RM and repetitions in reserve based resistance training approaches ranked between third and fifth, with moderate negative to trivial positive effects on strength, jump, and sprint performance.

A recent meta-analytical review (Liao et al., 2021) revealed similar effects of VBT and TRF on strength performance (MD = 3.03 kg; 95% CI: −3.55, 9.61; I^2^ = 0%), despite lower training volume during VBT approaches. Based on P-scores our findings revealed favorable effects of both high and low velocity loss based resistance training approaches (on strength performance) compared to traditional 1RM or RIR based resistance training approaches. Overall, traditional 1RM based resistance training to failure does not necessarily lead to higher strength gains (Izquierdo-Gabarren et al., 2009). In addition, recent findings revealed reduced training induced stress and less need of recovery via low velocity loss based resistance training (Held et al., 2021a). This finding has been confirmed by other scholars (Sánchez-Medina and González-Badillo, 2011), who observed a high correlation between increasing velocity loss and mechanical or metabolic stress. Furthermore, neuromuscular performance recovers faster post low velocity loss resistance training rather than post high velocity loss approaches (Pareja-Blanco et al., 2019).

In line with a previous systematic review (Włodarczyk et al., 2021), we observed (based on P-scores) favorable effects of low velocity loss approaches on jump performance adaptations. In this context, high velocity loss approaches seem to have negative effects on the type IIX muscle fibers (Pareja-Blanco et al., 2017a). While 8 weeks of low velocity loss based resistance training did not induce a reduction in type IIX fiber content (of the m. vastus lateralis), high velocity loss based resistance training produced a significant reduction in type IIX content (Pareja-Blanco et al., 2017a). In particular, the rate of force development dependent jump performance (McLellan et al., 2011; Laffaye et al., 2014; Maffiuletti et al., 2016) has been attributed at the proportion of IIX fibers (Andersen et al., 2010). Confirming these previous findings, Martinez-Canton and colleagues (Martinez-Canton et al., 2021) revealed increased CAMKII (calmodulin kinase II) activity *via* high velocity loss training, which has been linked to a decrease in IIX isoforms through a change in calcium handling (Tavi and Westerblad, 2011; Summermatter et al., 2012; Gehlert et al., 2015). Therefore, Pareja-Blanco and colleagues (Pareja-Blanco et al., 2017b) concluded in line with our findings that an optimal velocity loss ranges from 10 to 20% for jump performance developments.

While Pareja-Blanco and colleagues (Pareja-Blanco et al., 2017b) emphasize the benefits of low VL for sprint performance adaptations, a recent meta-analytic review (Liao et al., 2021) indicates comparable sprint performance adaptations at low and high velocity loss approaches (MD = 0.01 s; 95% CI: -0.06, 0.07; I2 = 0%). In contrast, the high velocity loss approach scores best in our P-score ranking. However, there is a large overlap in the variances of the low and high velocity loss approaches, which makes it difficult to clearly distinguish between them in terms of effectiveness. Nevertheless, our ranking shows a substantial gap between both low and high velocity loss approaches and RIR, VBT, and TRT, which again highlights the value of velocity loss based resistance training.

Apart from high P-scores, the low and the high velocity loss approaches revealed only small positive mean effects compared to a traditional resistance training approach. Furthermore, 95% confidence intervals revealed small negative (−0.42) to large positive (+1.26) strength, jump and sprint adaptation effects compared to a traditional resistance training approach. Nevertheless, considering the lower loads and the resulting reduced needs for recovery associated with lower velocity loss training (Sánchez-Medina and González-Badillo, 2011; Pareja-Blanco et al., 2019; Held et al., 2021a), these small effects are relevant for athletes and coaches in a short and long term perspectives on the training process. However, a corresponding sample size estimation for a randomized crossover trail (paired *t*-test; SMD = 0.41; α error = 0.05; power (1-β error) = 0.90). using G*Power (Version 3.1.9.6) (Faul et al., 2007) revealed a required sample size ≥50 participants, which is substantially above the mean sample size of the included studies of this network meta-analyses. Therefore, future research on velocity loss approaches should consider larger sample sizes or individualized approaches with more frequent repeated measures (Hecksteden et al., 2015, 2018). based on single-subject designs (Hecksteden et al., 2015, 2018) and/or Bayesian statistics using informative priors (Hecksteden et al., 2022) could tackle these sample sized related limitations (Hecksteden et al., 2021).

A limitation of the current network meta-analysis is that only 14 studies could be identified that met our inclusion criteria. These stringent inclusion criteria were necessary to achieve homogeneous networks. Furthermore, this decision is confirmed by the finding that our network models show no evidence of heterogeneity or inconsistency. Nevertheless, the used network meta-analytical approach enables effect size estimation based on both direct and indirect study comparison (Rücker and Schwarzer, 2015). Therefore, our results are in line with previous reviews and meta-analyses (Liao et al., 2021; Włodarczyk et al., 2021), despite the aforementioned limitations.

This network meta-analytical review revealed that (high and low) velocity loss based resistance training approaches revealed favorable effects on strength, jump, and sprint performance compared to other velocity based, traditional 1RM, and repetitions in reserve based resistance training approaches. In particular for jump performance, low velocity loss resistance training approaches induced favorable improvements compared to all other resistance training approaches. Overall, these findings indicate that the choice of resistance training approach and in particular the level of velocity loss has an impact on the magnitude of the effects and should, therefore, be carefully considered by coaches and athletes. In particular, velocity loss based approaches seem beneficial for increasing strength, jump and sprint performances. In particular, low velocity loss within a resistance training seems to be beneficial for strength and jump performance adaptations. In contrast, sprint performance seems to benefit from both low and high velocity loss based resistance training. In this context, future research should focus on the optimal amount of this velocity loss.

## Data Availability

The original contributions presented in the study are included in the article/Supplementary Material, further inquiries can be directed to the corresponding author.
